# Transcriptomic Analyses and Weighted Gene Co-Expression Network Analysis (WGCNA) Identify Key Drought-Responsive Genes in Rice Roots (*Oryza sativa* L.) Under PEG Treatment

**DOI:** 10.3390/plants15111591

**Published:** 2026-05-22

**Authors:** Shengjie Yan, Zining Jiang, Xue Liu, Yixuan Huang, Ni Li, Weiping Wang, Luis A. J. Mur, Zhi Liu, Dongyang Lei, Xianwen Zhang

**Affiliations:** 1 College of Agronomy, Hunan Agricultural University, Changsha 410128, China; m15274582775@163.com; 2 College of Bioscience and Biotechnology, Hunan Agricultural University, Changsha 410128, China; sx20250460@stu.hunau.edu.cn (Z.J.); lx11231998@163.com (X.L.); m18807424252@163.com (Y.H.); tigerzhiliu@126.com (Z.L.); 3 National Key Laboratory of Hybrid Rice, Hunan Hybrid Rice Research Center, Changsha 410125, China; lini@hhrrc.ac.cn (N.L.); wangweiping@hhrrc.ac.cn (W.W.); 4 Department of Life Sciences, Aberystwyth University, Wales SY23 3DA, UK; lum@aber.ac.uk

**Keywords:** drought, rice, root, RNA-seq, WGCNA, yeast, candidate genes

## Abstract

Rice depends on its root system to perceive drought, a major environmental constraint that leads to severe yield losses worldwide. To dissect the underlying molecular basis, we conducted a comparative analysis of drought-sensitive (WAB) and drought-tolerant (IR65) rice genotypes that exhibited divergent drought tolerance at the seedling stage. After exposure to 15% PEG6000 (−0.4 MPa) for two days, the shoot and root architectural traits of IR65 were better than those of WAB seedlings. Measurements of physio-biochemical parameters (SOD, CAT, POD, APX, H_2_O_2_, and proline) suggest that IR65 seedling roots exhibit greater ROS scavenging and osmotic adjustment capacity than WAB, aligning with tolerance to PEG-induced water deficiency. Transcriptomic assessments of roots identified 802 commonly differentially expressed genes (DEGs) during the drought time course (12, 24, and 48 h) in WAB and IR65. They were clustered into eight groups based on their expression profiles and mainly enriched in phytohormone signaling, protein phosphorylation, and transcription factors. Using weighted gene co-expression network analysis (WGCNA), nine significant modules were identified based on *n* = 382 of the DEGs. A total of 12 DEGs up-regulated in IR65 were distributed in five modules, and five of them were selected for rapid functional validation through in vivo yeast expression. The results showed that transgenic yeasts were tolerant to simulated drought conditions (135 mM PEG3350), indicating that these genes would be potential targets for rice improvement in drought tolerance in the future.

## 1. Introduction

Rice (*Oryza sativa* L.) is one of the most agronomically and economically important crops, being a staple food for over half of the world’s population, especially in developing countries [1]. In recent years, improvements in rice productivity have been hindered by climate change, as changes in the global water-cycling system are impacting agricultural production. Indeed, over one-third of the world’s total cultivated area is prone to reduced water availability [2]. Drought can affect plant growth from seed germination to the reproductive stages, stunting growth, reducing flowering efficiency and grain formation, and causing yield loss. In extreme cases, drought can lead to the death of rice plants [3,4]. To improve rice productivity, water resource management and agronomic irrigation practices are both essential to protect rice plants from drought stress [5]. As an additional strategy, identifying or generating rice germplasm with improved drought tolerance is important to improve worldwide food safety [6,7,8].

Rice has evolved complex resistance mechanisms to adapt to water-limited conditions, ensuring survival and reproduction [2,9,10]. Research into this area has identified large numbers of genes related to drought stress regulation, but genes linked to growth stage-specific effects in rice still need to be more extensively characterized [11,12]. Once identified, such genes can be effectively exploited in rice breeding programs. In the past decade, quantitative trait locus (QTL) mapping, marker-assisted selection (MAS), elite allele mining, and genetic transformation have significantly improved drought-related traits, facilitating the process of improved crop variety generation [6,13,14,15,16]. Furthermore, high-throughput RNA sequencing (RNA-seq) has emerged as a valuable technology to analyze newly developed rice varieties under drought [17,18,19,20,21,22,23]. Recent studies have used RNA-seq and weighted gene co-expression network analysis (WGCNA) to dissect genetic networks of various crops in response to drought stress and ease the identification of potential candidate genes [24,25,26,27].

Roots serve as fundamental organs that absorb water and nutrients from the soil. Importantly, they are an early sensor of water deficiency and enable rice plants to survive and sustain growth in dry fields [28]. Therefore, characterization of morphological and physio-biochemical changes in plant root systems under drought can provide critical criteria for screening drought-tolerant germplasm resources [29,30]. In wheat, RNA-seq and WGCNA methods were combined to identify a key regulatory module and potential functional genes linked to a root salt-tolerance phenotype [31].

One limitation to the rapid exploitation of any likely drought-tolerance gene is the time taken to fully characterize it in plants. An alternative approach is to characterize any gene function in a highly tractable, rapid-cycling system. Yeast (*Saccharomyces cerevisiae*) is a well-established unicellular model organism that offers a platform for the functional screening of plant-derived stress-tolerance genes [32]. Based on transcriptome sequencing analyses of ryegrass under heat exposure, a yeast screening system revealed that genes encoding ribosomal proteins may play a critical role in the response to high-temperature stress [33]. Overexpression of a lysine dehydrogenase from the straw mushroom (*Volvariella volvacea*) conferred yeast a cold-stress-tolerant phenotype, suggesting that enhanced lysine metabolism may improve cold tolerance in the straw mushroom [34]. The FOX (full-length cDNA over-expressor) hunting system identified a series of salt-tolerance genes in *Tamarix hispida* [35], wild emmer wheat [15], and beach morning glory (*Ipomoea pes-caprae*) [32]. Additionally, a genome-wide analysis of the ERF transcription factor family in *Melilotus albus*, combined with a yeast sensitivity assay, identified four drought-tolerance functional genes [36]. These findings provide potential targets for genetic engineering approaches to improve abiotic stress resistance in plants.

Up to now, numerous studies have characterized drought-tolerance mechanisms of different species using comparative transcriptome analyses, whereas root-specific temporal response profiles are relatively fragmented in rice. In our previous research, two contrasting rice genotypes (WAB and IR65) were targeted as having different levels of drought resistance based on a comprehensive analysis of 26 genotypes at germination and seedling stages under PEG-simulated drought conditions [37]. In this current study, morphological and physio-biochemical indexes of WAB and IR65 roots at the seedling stage were measured under drought. The differences in drought response mechanisms of the roots of the two rice plants were further explored using RNA-Seq. WGCNA was used to associate divergent drought phenotypes with gene expression patterns, and significant co-expression modules were identified. DEGs from the key modules were then characterized via yeast overexpression under PEG treatment. The aim of this study was to dissect the divergent drought response mechanisms between contrasting rice genotypes and mine potential genetic resources for rice improvement in drought tolerance, which paves the way for breeding water-saving rice lines for practical use in agriculture.

## 2. Results

### 2.1. Root Phenotype of Two Contrasting Rice Genotypes Under Drought Stress

**Figure 1:**
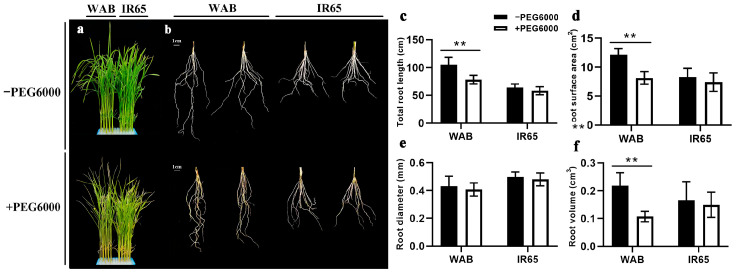
Phenotypic analyses of WAB and IR65 rice plants after 48 h of 15% PEG6000 (+PEG6000) treatment compared to untreated controls (−PEG6000). (**a**) Shoot phenotype of WAB and IR65 two-week-old seedlings. (**b**) Root phenotype of WAB and IR65 two-week-old seedlings, bar = 1 cm. (**c**) Total root length (TRL). (**d**) Root surface area (RSA). (**e**) Root diameter (RD). (**f**) Root volume (RV). The standard error line represents the standard deviation (SD) (*n* = 10). Statistically significant differences are indicated by ** *p* < 0.01 through one-way ANOVA.

After administering a 15% PEG6000 treatment for 48 h, the growth of both types of rice seedlings was inhibited by drought stress. The leaf tips of the drought-sensitive rice WAB significantly curled and yellowed as compared to those of IR65 (Figure 1a). Root diameter (RD) of both rice genotypes was relatively less affected by drought stress, declining by 5.61% in WAB and 2.09% in IR65 compared to their controls. In contrast, total root length (TRL), root surface area (RSA), and root volume (RV) showed substantial reductions in WAB, with decreases of 27.32%, 32.46%, and 46.53%, respectively. These three traits in IR65 separately dropped by only 10.09%, 11.51%, and 9.85% (Figure 1b).

### 2.2. Physio-Biochemical Changes in the Roots of Two Contrasting Rice Genotypes Under Drought Stress

**Figure 2:**
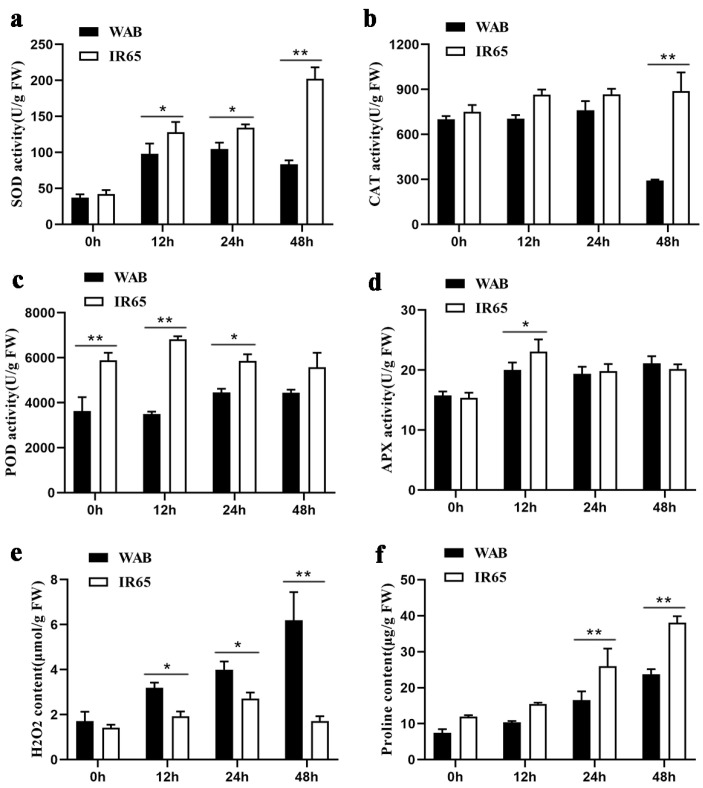
The effects of drought treatment (15% PEG6000) on physiological indexes in the roots of WAB and IR65 over a 48 h period. (**a**) Superoxide dismutase (SOD) activity. (**b**) Catalase (CAT) activity. (**c**) Peroxidase (POD) activity. (**d**) Ascorbate peroxidase (APX) activity. (**e**) Hydrogen peroxide content (H_2_O_2_). (**f**) Proline content. The standard error line represents the standard deviation (SD). Statistically significant differences are indicated by * *p* < 0.05 or ** *p* < 0.01 through one-way ANOVA. The bars represent SD (*n* = 3).

Under drought stress, IR65 roots exhibited consistently higher superoxide dismutase (SOD) activity than WAB across all time points, with increases of 30.36%, 28.48%, and 142.78% at 12 h, 24 h, and 48 h, respectively (Figure 2a). Catalase (CAT) activity did not differ significantly between the two genotypes except at 48 h, where IR65 showed higher activity than WAB (Figure 2b). POD activity in IR65 roots, which had a 61.18% higher basal level than WAB under well-watered conditions, peaked at 12 h of drought before declining to baseline, suggesting a rapidly responsive peroxidase system (Figure 2c). Ascorbate peroxidase (APX) activity in IR65 increased by 50.30% at 12 h relative to 0 h, but no differences were observed at other time points (Figure 2d). H_2_O_2_ content was 1.65-, 1.47-, and 3.60-fold higher in WAB than in IR65 during 12–48 h of drought, whereas levels were comparable before treatment (Figure 2e). Compared to 0 h, proline concentrations slightly increased in IR65 roots at 12 h but rose significantly by 117.46% and 218.43% at 24 h and 48 h, respectively, indicating an osmoprotective response that may complement enzymatic antioxidant defense (Figure 2f).

### 2.3. Transcriptomic and Correlation Analyses of the Responses of the Rice Genotypes to Drought

RNA-seq analyses were conducted on WAB and IR64 seedlings at 0 h, 12 h, 24 h, and 48 h following 15% PEG6000 treatment and from untreated controls. A total of 148.15 Gb clean reads were obtained from the raw data filtered by quality control. The maximum value for Q30 reached 94.13%, whereas the GC content was between 50.70% and 51.65%. The quality-controlled clean reads for each sample were aligned with the reference genome, and the efficiency of mapping ranged from 90.48 to 95.25 (Table S1). The outcome of RNA-seq indicates that these high-quality data were suitable for subsequent bioinformatics analyses. FPKM (fragments per kilobase of transcript per million fragments mapped) values of each sample were normalized against the control (CK). The overall distribution of gene expression across all samples was similar, and most of those genes exhibited FPKM values concentrated primarily between 1 and 100 (Figure S1a). The overall boxplot for FPKM indicates that there were no significant deviations in gene expression distribution among different samples (Figure S1b). The principal component analysis (PCA) showed that the distances between biological replicates were minimal, while the distances among different treatments were greater (Figure S1c). The correlation analyses performed using gene expression data revealed a high degree of correlation among biological replicates, with Pearson correlation coefficients (r^2^) above 0.98. The biological replicates clustered within the same branch, indicating a strong reproducibility of the experimental results (Figure S1d).

### 2.4. Targeting Drought-Responsive Differentially Expressed Genes (DEGs)

|Log_2_Fold Change| ≥ 1 and false discovery rate (FDR) < 0.05 were set as the inclusion criteria for screening DEGs. The number of DEGs at 12 h, 24 h, and 48 h was determined for each genotype in drought-stressed roots. In total, 4450 DEGs (2725 up-regulated and 1725 down-regulated), 4680 DEGs (2613 up-regulated and 2067 down-regulated), and 5093 DEGs (2975 up-regulated and 2118 down-regulated) were identified in WAB roots during 12 h, 24 h, and 48 h of drought, respectively (Figure 3a). In IR65, 5662 DEGs (2696 up-regulated and 2966 down-regulated), 5927 DEGs (2945 up-regulated and 2982 down-regulated), and 6929 DEGs (3339 up-regulated and 3590 down-regulated) were identified over three timepoints of drought conditions (Figure 3a). The number of DEGs in IR65 roots for each time point was higher than that in WAB. When DEGs were compared using a Venn diagram, 802 DEGs were common in WAB and IR65 during the two days of drought treatment, likely including genes that were crucial to the drought response (Figure 3b). The 802 DEGs could be partitioned into eight distinct co-expression clusters of varying membership sizes (Figure 3c,d). To validate the RNA-seq data, nine DEGs were randomly selected and their relative expression levels determined by qRT-PCR analyses (*n* = 3 replicates). *Actin* (GenBank accession number: NM_001418593) was used as the reference gene. The qRT-PCR determined expression of all of the nine DEGs was consistent with RNA-seq data, although their absolute expression levels were lower, with the exception of three genes (*LOC4339461*, *LOC4327611*, and *LOC4327485*) in IR65 (Figure 3e).

### 2.5. GO and KEGG Enrichment Analyses of DEGs

A total of 1228, 1159, and 1362 DEGs in WAB were assigned to the top 20 GO categories at 12 h, 24 h, and 48 h of drought stress, respectively (Figure S2). For IR65, 1356, 1348, and 1457 DEGs were mapped to the top 20 GO categories (Figure S3). In both rice genotypes, GO:0006979 (response to oxidative stress), GO:0042744 (hydrogen peroxide catabolic process), and GO:0006749 (glutathione metabolic process) were the top three GO terms in the biological process (BP) category. GO:0048046 (apoplast) and GO:0005576 (extracellular region) were the top two GO terms in the cellular component (CC) category. GO:0020037 (heme binding), GO:0043565 (sequence-specific DNA binding), GO:0005506 (iron ion binding), and GO:0004497 (monooxygenase activity) were the top four GO terms in the molecular function (MF) category (Figures S2 and S3). Accordingly, all DEGs were assigned to the top 20 KEGG pathways related to metabolism (ME), environmental information processing (EIP), and organismal systems (OS). Considering differential expression as discrete timepoints, in WAB, there were 539, 618, and 616 DEGs at 12 h, 24 h, and 48 h of drought stress, respectively (Figure S4). For IR65, 662, 705, and 764 DEGs at 12 h, 24 h, and 48 h of drought stress, respectively, could be linked to KEGG pathways (Figure S5). Most DEGs mapped to ko00940 (phenylpropanoid biosynthesis), ko04626 (plant–pathogen interaction), ko04075 (plant hormone signal transduction), and ko04016 (MAPK signaling pathway—plant) (Figures S4 and S5).

### 2.6. Comparative Analyses of DEGs Between WAB and IR65 Roots Under Drought

**Figure 4:**
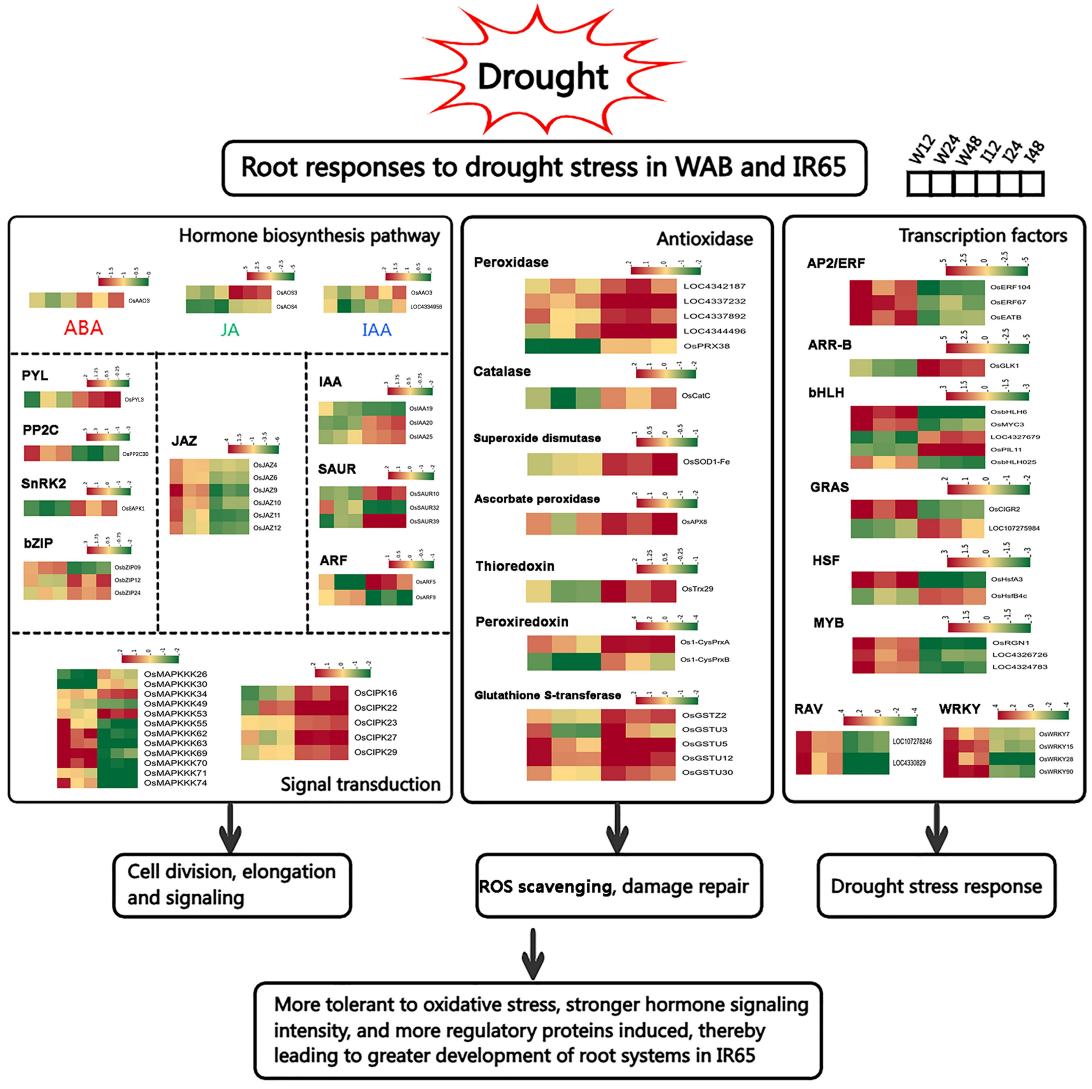
Mechanistic diagram of the root responses of WAB and IR65 to drought stress.

Comparative expression profiles of drought-responsive DEGs over a 48 h period were analyzed through pathway-specific heatmaps: hormone synthesis and signaling, antioxidative stress, kinase signaling cascades, and transcriptional regulation (Figure 4). In IR65, genes positively regulating hormone (ABA, JA, and IAA) response pathways were significantly up-regulated, while negative regulatory genes were down-regulated. The expression of most *MAP3K* genes was inhibited by drought in IR65 but induced in WAB. A converse pattern was seen with five *CIPK* genes, which were up-regulated in IR65. Some DEGs encoding antioxidative enzymes were obviously up-regulated with drought, but expression levels were higher in IR65 than in WAB. A total of 22 genes from eight transcription factor families showed divergent expression patterns between WAB and IR65, with most genes being down-regulated in IR65.

Under drought stress, hormone-, kinase-, antioxidase-, and transcription factor-related genes showed divergent expression between WAB and IR65 roots. IR65 exhibited better drought stress adaptation by up-regulating or down-regulating these genes.

### 2.7. WGCNA-Based Identification of Significant Co-Expression Modules and Module Visualization

**Figure 5:**
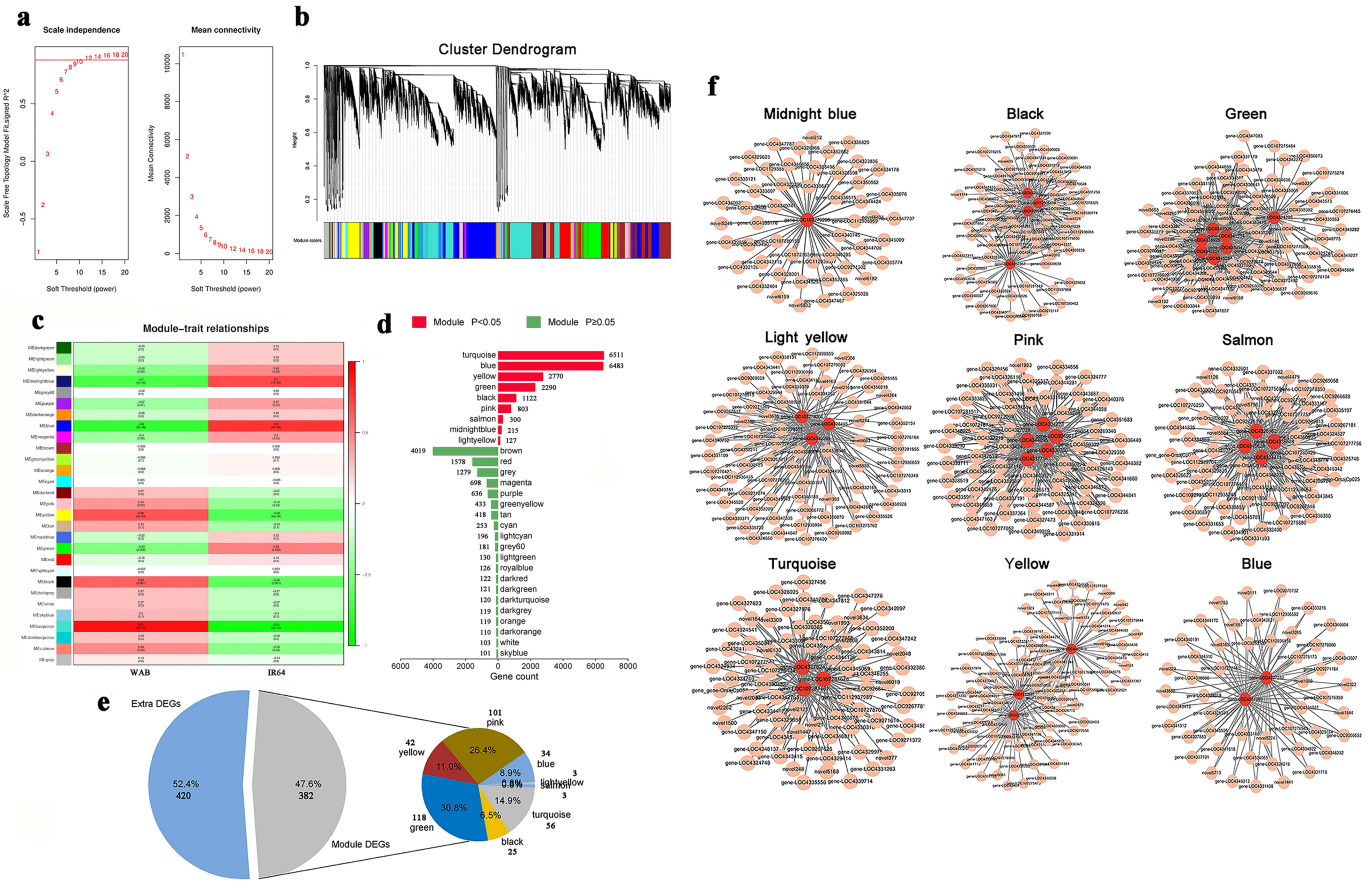
WGCNA targeting of key hub genes in the responses of rice roots to drought. (**a**) Scale-free topology model and mean connectivity. (**b**) Hierarchical cluster tree identified by WGCNA. In the dendrogram, the different colors below the branches indicate distinct co-expression modules. (**c**) Module–trait correlations and corresponding *p*-values. The numbers in each cell represent the correlation coefficients and correlation significance levels (in parentheses). Cell color intensity reflects the magnitude of the correlation. (**d**) Statistics of gene number in each module. Red bars represent statistically significant associations (*p* < 0.05), and green bars denote non-significant relationships. (**e**) Distribution of differentially expressed genes (DEGs) in each module with high correlation. (**f**) Visualization of highly related genes in different modules. Red-colored nodes suggest their central role in the network.

A total of 31,483 genes with an FPKM ≥ 1 were used for WGCNA. Using scale independence and mean connectivity, genes gathered in the same cluster showed high interconnection and correlation coefficients (Figure 5a). Analyses suggested 29 distinct co-expression modules, shown as branches of the dendrogram through the dynamic tree cut method, each labeled with a unique color (Figure 5b). There were nine significant *(p*-value < 0.05) co-expression modules (Figure 5c). Among these modules, four exhibited positive correlations with drought stress, while the remaining five showed negative correlations. Figure 5d shows the number of DEGs in the co-expression modules (in red), while those in non-differential modules are shown in green. The turquoise and blue modules exhibited the highest gene enrichment, containing 6511 and 6483 genes, respectively (Figure 5d). Among the 802 drought-responsive DEGs common to both genotypes, nearly half were assigned to nine separate, significant co-expression modules, with the pink (26.4%) and green (30.8%) modules being the most predominant (Figure 5e). To identify the hub genes in these modules, the co-expression network was analyzed and visualized by Cytoscape based on their intramodular connectivity (kME) (Figure 5f). The top genes in each module visualized as hubs were highlighted in red, while their highly interconnected partners were marked in light red. A total of 32 center genes were identified among nine closely related modules of midnight blue, blue, light yellow, yellow, pink, green, black, turquoise, and salmon. Genes encoding membrane-anchored kinases, like *LOC107279296*, *LOC4351086*, *LOC4339142* (*OsRPK1*), *LOC4345707* (*OsPEPR1*), *LOC112936119*, *LOC107281678*, *LOC4327824*, and *LOC4324411*, were often found in these modules. *LOC4337232* and *LOC9269614,* located in the blue and pink modules, respectively, are related to the maintenance of redox status. The gene products of *LOC4330360*, *LOC4335919*, and *LOC4324295*, which belonged to the pink, yellow, and green modules, were annotated as ubiquitin ligases.

### 2.8. Screening of Drought-Responsive DEGs for Validation by Yeast

**Figure 6:**
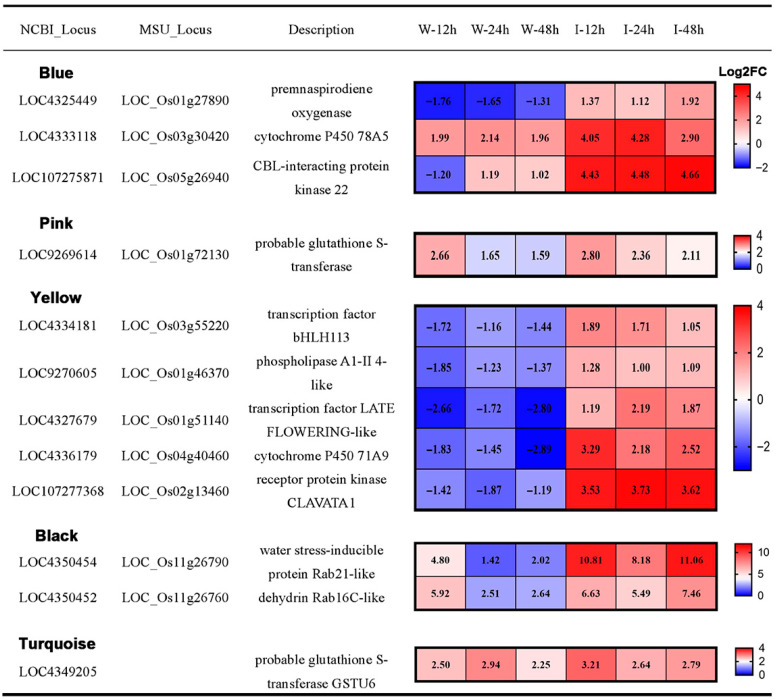
Twelve differentially expressed genes (DEGs) selected from five significant modules by WGCNA based on expression levels in WAB and IR65.

**Figure 7:**
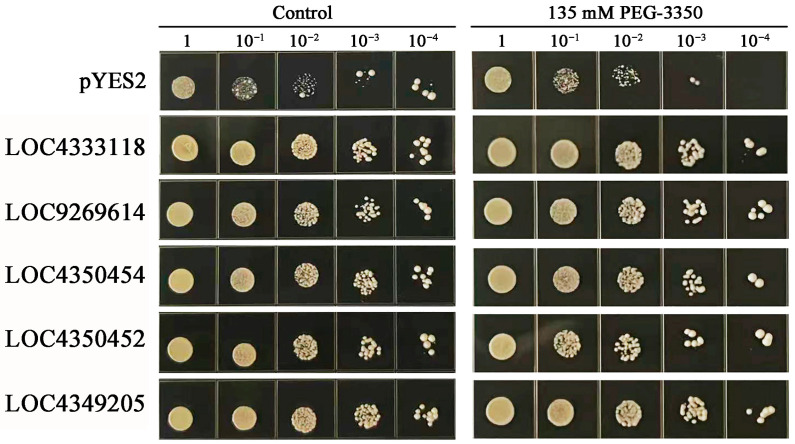
The PEG3350 tolerance test of five candidate rice genes following heterologous expression in yeast. Yeast cultures (OD_600_ to 0.2) were serially diluted to OD_600_ values of 10^−1^, 10^−2^, 10^−3^, and 10^−4^, and yeast liquid was spotted onto solid SG-Ura plates without or with PEG3350 (135 mM). pYES2, as a negative control group, was transformed with the empty vector, and five transgenic yeasts were treated as experiment groups.

To further verify whether DEGs from significant co-expression modules are potential functional candidates based on RNA-seq and WGCNA, 12 DEGs assigned to five significant co-expression modules (blue, pink, yellow, black, and turquoise) were selected. Five hub genes, cytochrome P450 (*LOC4333118*), two glutathione S-transferases (*LOC9269614* and *LOC4349205*), and two dehydrin proteins (*LOC4350452* and *LOC4350454*), with kME values ranging from 700 to 1200 in the WGCNA network and up-regulated in both WAB and IR65 roots or exclusively up-regulated in IR65 under drought, were taken forward for functional analysis using heterologous yeast expression (Figure 6). Expression of each gene in yeast allowed greater colony growth, compared to the empty plasmid with PEG treatment (Figure 7). These observations suggest that DEGs are potential candidates for subsequent drought-tolerance validation in rice.

## 3. Discussion

Water scarcity is often a feature of variable natural environments and poses a significant threat to global crop productivity [38]. This has initiated considerable efforts focusing on screening and identifying water-saving germplasm resources across various crop species. The root is a key organ that perceives the initial signals of water deficiency [39]. Modulation of root system architecture (RSA) has emerged as an essential strategy against low humidity conditions by altering root density and promoting a deeper root system to access the water-sufficient soil layers [40,41]. This study investigated and compared the morphological, physio-biochemical, and molecular responses of the roots to drought stress between two rice genotypes with different degrees of drought tolerance under PEG-simulated drought conditions. An integrated analysis of transcriptomic data and WGCNA was employed to target key drought-responsive genes. Crucially, we used a heterologous expression system in yeast to suggest that they could confer increased tolerance under water-stressed conditions. The targeted genes and, indeed, our method for rapid functional screening could be exploited in breeding drought-resilient rice genotypes.

In this study, polyethylene glycol (PEG) 6000 was used as an osmotic agent to simulate drought stress. While PEG is widely employed for this purpose, it is important to acknowledge its potential physicochemical interactions with plant metabolites, particularly phenolic compounds, which could influence the interpretation of our physiological and biochemical assays [42,43]. Thus, based on our PEG treatment conditions, interactions between PEG6000 and phenolic compounds cannot be excluded. Despite this, both the drought-tolerant IR65 and the sensitive WAB were treated under identical PEG conditions; any systematic bias from PEG-phenol interactions would equally affect the two genotypes.

Similar comparative analyses to ours, involving total antioxidant capacity, polyphenol oxidase (PPO), and phenylalanine ammonia lyase (PAL), were assessed in roots of different poplar genotypes under PEG6000 treatments [44]. Moreover, the transcriptional changes identified by RNA-seq, which serve as the principal evidence for our analyses, remain independent of any direct chemical interactions between PEG and phenolic compounds. Future work using soil-drought systems will be required to further validate these findings without the potential effects introduced by PEG. The 15% PEG6000 concentration, on the other hand, has been validated as the optimal level for drought-tolerance screening in rice at the seedling stage, as it maximizes genotypic discrimination while avoiding excessive stress that would compromise physiological and molecular measurements [45,46].

### 3.1. IR65 Roots Are Less Susceptible to Drought than Those of WAB

Characterization of root system parameters under PEG6000-simulated drought stress (Figure 1a–f) showed that root shrinking was prominent in WAB, whereas IR65 exhibited the opposite pattern. Findings in a range of species [47,48,49] suggest that this was consistent with a more efficient water retention capacity in IR65. This root phenotype also aligned with physio-biochemical measurements under drought. Thus, while there were increased activities of detoxifying enzymes, such as CAT, POD, SOD, and APX, in both genotypes, these were greater in IR65 roots (Figure 2a–d). Maintenance of such redox systems is integral for cellular homeostasis, particularly after exposure to adverse factors [50], and is linked to lower ROS levels in drought-tolerant plants [51,52]. Generally, endogenous H_2_O_2_ levels are negatively correlated with antioxidant enzyme activity [53]. It was notable that H_2_O_2_ levels were higher in WAB than in IR65 roots during drought stress, particularly at 48 h (Figure 2e). This suggested the effectiveness of H_2_O_2_ scavenging in IR65 to reduce oxidative damage in root cells. Proline acts as a key organic osmolyte that sustains cellular turgor under osmotic stress [54] and also functions as a critical mediator of the oxidative defense system [55]. In our experiments, as drought progressed, proline accumulated in higher levels in IR65 compared to WAB (Figure 2f), further suggesting that root cells in IR65 undergo less oxidative damage and counteract adverse osmotic pressure under continuous drought. There is a positive correlation between grain yield and proline content under drought-stressed conditions in wheat [56], and our data suggests that this could be used to select drought-tolerant rice genotypes.

### 3.2. Patterns of Transcriptional Reprogramming in IR65 Roots Suggest Better Drought Adaptation

The molecular mechanisms underlying drought response in plants encompass intricate genetic networks and highly complex regulatory processes. Through RNA-seq technology, differences in drought-responsive expression patterns in the roots of the two rice genotypes were defined (Figure 4). DEGs from IR65 roots showed greater enrichment of drought-responsive genes at each time point compared to those from WAB. This suggests that wider resources are dedicated to drought responses in drought-tolerant germplasm.

Phytohormone-mediated signal transduction events contribute to drought-adaptive responses by modulating the expression of downstream stress-responsive genes [57]. The drought-responsive ABA signaling pathway is principally mediated by an integrated PYLs-PP2Cs-SnRK2s-ABFs regulatory network. IR65 expression patterns suggest greater ABA responsiveness in its roots. IR65 roots exhibited a relatively elevated expression of *OsPYL3* and *OsSAPK1*, two core components of ABA signaling, compared to WAB. Overexpression of the ABA receptor gene *OsPYL3* can increase drought stress tolerance in rice [58]. The responsiveness of *OsSAPK1* to ABA suggests a potential role in drought response regulation [59,60]. The expression of *OsPP2C30*, a negative regulator of the ABA signaling pathway [61], was significantly lower in IR65 than in WAB. This could reduce any inhibitory in vivo interaction between OsPP2C30 and OsPYL3 in the presence of ABA [58]. ABA signaling may also stimulate auxin production in plant roots [62]. IAA biosynthesis gene expression was elevated in the roots of IR65 in response to drought, suggesting that the maintenance of root system growth could also involve enhanced IAA signaling. Transcription of *OsIAA20* was up-regulated in IR65. Interestingly, overexpression of *OsIAA20* conferred drought tolerance in rice and activated the expression of ABA-induced genes, such as *OsRab21* [63], indicating an interplay between IAA and ABA signaling in drought response. Crosstalk between ABA and JA has also been documented as a regulatory mechanism in drought adaptation [64,65,66]. JAZ proteins are repressors of JA signaling [67], and this was consistent with OsJAZ1 acting as a transcriptional regulator suppressing the activation of drought-adaptive responses [68]. In line with this, the transcription of several *JAZ* genes was significantly decreased in IR65 but was up-regulated in WAB throughout the entire drought treatment period (Figure 4). This suppression of JAZ proteins may be important to exert function in JA-mediated drought tolerance in IR65.

Kinase-mediated signaling cascades orchestrate the integration of downstream adaptive responses to environmental stresses. We found that most of the *MAPKKK* genes were silenced in IR65, while five *CIPK* genes were induced under drought conditions (Figure 4). Although MAPKKKs are recognized as positive regulators of drought responses [69,70], suppressive roles have also been documented [71,72,73]. In particular, OsMAPKKK63 negatively regulates salt stress responses [74]. Our study suggested that, at least in part, the positive roles for kinases in the drought response were dominated by CIPKs, activated by stress-induced Ca^2+^ sensors (calcineurin B-like proteins) [75].

An integrated network of redox-responsive mechanisms undoubtedly enables plants to adapt to diverse stress conditions [76]. This appeared to be a feature of the IR65 root system (Figure 4), which was reflected in its efficient ROS scavenging capacity (Figure 2a–f). One class of antioxidant genes of particular importance could be peroxiredoxins, which can scavenge H_2_O_2_, organic hydroperoxides, and peroxynitrite. *Os1-CysPrxB* is root-preferentially expressed in rice [77] and in IR65, suggesting a role in countering the stress-induced redox imbalance (Figure 4). Transcription factors are also crucial integrators of drought responses, and several were more highly expressed in WAB than in IR65 under drought. MYB, RAV, WRKY, and AP2/ERF families are consistently identified as key regulators of drought tolerance. Several rice MYB genes, including *OsMYB2* and *OsMYB4*, have been shown to enhance drought tolerance when overexpressed, largely through improved osmotic adjustment and increased expression of stress-responsive genes [78]. *OsWRKY11*, *OsWRKY30,* and *OsWRKY47* have been shown to enhance drought tolerance by activating protective genes and improving antioxidant capacity [79,80]. The AP2/ERF superfamily, including the DREB and ERF subfamilies, plays vital roles in drought responses. OsDREB1A and OsDREB2A enhance drought tolerance through the transcriptional activation of LEA proteins and dehydrins [81]. The prominence of these drought-tolerance factors in WAB indicates the existence of genotype-specific responses to drought. In WAB, these responses could be compromised by the induction of RAV proteins, as overexpression of OsRAV9 compromised drought tolerance by repressing *OsbZIP23* [82]. In IR65, the B-type response regulators (ARR-B) appeared to be more prominent. A recent study showed that OsRR41 was essential for maintaining ionic balance, ROS detoxification, and stress-responsive gene expression under salinity stress [83]. This would also be relevant to drought, and clearly, ARR-B requires more extensive study within the context of drought tolerance in rice.

### 3.3. Functional Verification of the Potential Candidate Genes by Yeast System

To highlight key regulatory nodes, WGCNA was employed to advance our understanding of the underlying mechanisms and guide future breeding strategies. Thus, WGCNA was used to identify key co-expression modules containing DEGs (Figure 5 and Figure 6). With these modules, five genes involved in drought responses were targeted. Importantly, we employed a rapid screening approach based on transgenic yeast to suggest their ability to confer osmotic tolerance. In each case, the over-expressed genes appeared to improve the tolerance of the yeast to PEG-induced osmotic stress (Figure 7). The likely mechanisms of the selected genes were suggested from consulting the wider literature. Cytochrome P450s (CYPs) constitute a large protein family that mediates diverse physiological processes, primarily by catalyzing the biosynthesis of secondary metabolites and participating in detoxification pathways [84], and these are likely to be functioning to confer osmotic stress tolerance in our study. Overexpression of *GmCYP* in transgenic tobacco, encoding a soybean cytochrome P450 enzyme, has been shown to improve tolerance to drought stress [85]. Other genes have better characterized roles in drought tolerance. Dehydrins (DHNs) are highly hydrophilic proteins that play diverse protective roles in plant cells under drought stress [86], and ectopic expression of *DHN* genes confers enhanced drought tolerance in plants [32,87,88]. As major detoxifying enzymes, glutathione S-transferases (GSTs) are critically involved in modulating plant responses to both abiotic and biotic stresses [89]. GSTs play a pivotal role in mitigating the negative effects of drought-stressed plants through removing excessive ROS [90,91,92]. These drought-responsive genes validated in this study via yeast assays could be used as candidate targets in rice breeding programs.

### 3.4. Future Perspectives

Rice plants adjust their root system architecture to adapt to water deficit stress by orchestrating intricate molecular networks. However, drought is a polygenic trait characterized by low heritability and significant environmental influence. To overcome this, comparative analyses of root transcriptome profiles have been adopted for a decade to unravel the genetic and physiological basis of the differences between rice resources with differential drought tolerance [18,22,93,94]. In this study, transcriptomic analyses revealed distinct gene expression profiles between the drought-sensitive rice WAB and the drought-tolerant germplasm IR65. Interestingly, WAB displayed longer roots than IR65, suggesting a potential trade-off, wherein WAB prioritizes carbon allocation to root elongation at the cost of drought tolerance. This phenotype may result from pleiotropic effects of root architecture genes inherent to WAB that negatively interact with drought response pathways. However, due to the substantial genetic background differences between these two rice plants, analyzing expression patterns alone is insufficient to elucidate the genetic basis underlying these phenotypic differences.

To complement these transcriptomic analyses, establishing a structured breeding population derived from a WAB × IR65 cross would support a systematic identification of drought-tolerance genes and accelerate the improvement of associated agronomic traits. At present, developing introgression lines or near-isogenic lines (NILs) from elite parents through successive backcrossing provides a robust approach to identify functional genes associated with root system architecture (RSA) and drought tolerance. This strategy effectively minimizes genetic background interference, enabling more precise dissection of target traits. When high efficiency and accuracy in gene discovery are required, forward genetic approaches, such as genome-wide association studies (GWASs) and bulked segregant analyses (BSAs), facilitate fine-mapping and cloning of quantitative trait loci (QTLs) that govern desirable root traits in rice. Additionally, comprehensive genome-wide resequencing of both parents could enable the detection of genome-wide, single-nucleotide polymorphisms (SNPs) and insertions and deletions (InDels), providing valuable molecular markers for dissecting natural variation and supporting molecular breeding efforts. Given the current limitations in applying transgenic approaches for developing novel rice germplasms with enhanced drought tolerance, mining QTLs and molecular markers associated with root traits remains a promising strategy to ensure stable rice production.

## 4. Materials and Methods

### 4.1. Plant Materials and Experiment Design

Two rice samples were provided by Hunan Hybrid Rice Research Center, Changsha, China: *Oryza sativa* L. ssp. japonica, WAB 878-6-37-5-1-P1-HB (WAB), which has light leaves and a weak tolerance to drought, and *Oryza sativa* L. ssp. Indica IR 65610-38-2-4-2-6-3 (IR65), which has rough leaves and a strong tolerance to drought. Yoshida nutrient solution was employed to cultivate the seedlings in climatic chambers (28 °C/25 °C with 16 h (day)/8 h (night), an illumination intensity of 450 µM m^−2^ s^−1^ and 80% relative humidity). Two-week-old seedlings were subjected to the Yoshida nutrient solution containing 15% PEG6000 (purity ≥ 99.0%, Solarbio, Beijing, China) to simulate osmotic shock. The root tissues of seedlings with similar growth vigor in the shoot were selected and treated for 0 h, 12 h, 24 h, and 48 h with 15% PEG6000, followed by physiological and transcriptomic analyses.

### 4.2. Root Measurements

The root system architecture of 10 rice plants randomly selected from the CK (0 h) and experimental groups treated with 15% PEG6000 for 48 h was scanned using an EPSON1680 scanner (Epson, Long Beach, CA, USA) and was analyzed using WinRHIZO software (WinRHIZO Pro2004, version 5.0; Regent Instruments Inc., Quebec, ON, Canada) to measure total root length, root surface area, root diameter, and root volume, respectively. For root-background distinction, the automatic threshold function of WinRHIZO was applied to discriminate roots from the background for improved reproducibility. Diameter class boundaries were set from 0.1 to 1.9 mm with 0.1 mm increments, based on the typical root diameter range of rice seedlings.

### 4.3. Measurement of Physio-Biochemical Indices

Fresh root samples (approximately 0.1 g) were ground in liquid nitrogen and homogenized in precooled PBS. After centrifugation, the supernatant was collected for physiological assays. The activities of catalase (CAT), superoxide dismutase (SOD), ascorbate peroxidase (APX), and peroxidase (POD), as well as the contents of hydrogen peroxide (H_2_O_2_) and proline, were determined using specific assay kits (Solarbio, Beijing, China) in accordance with the manufacturer’s protocols. The concentrations of H_2_O_2_ and proline were calculated based on standard curves following the manufacturer’s instructions.

### 4.4. RNA Extraction, Library Construction, and Transcriptome Sequencing

Total RNA from the 24 samples was isolated using TRIzol reagent (Tiangen, Beijing, China). The high-quality total RNA per sample was used as input material for the RNA sample preparations. The cDNA sequencing libraries were constructed using an NEBNext Ultra RNA Library Prep Kit for Illumina (NEB, Ipswich, MA, USA). In total, 24 library preparations were sequenced on the DNBSEQ platform to synthesize 150 bp paired-end reads (BGI Genomics, Shenzhen, China).

### 4.5. Quality Control and Alignment of RNA-Seq Reads

Raw data in fastq format were first processed using in-house perl scripts. Clean reads were obtained by eliminating adaptor sequences, unknown sequences ‘N’, and low-quality reads from raw data. Q20, Q30, GC content, and sequence duplication levels of the clean data were calculated (Table S1). All analyses were based on high-quality, clean reads. Clean reads from all samples were mapped to the reference genome for the rice cultivar Nipponbare (https://ftp.ncbi.nlm.nih.gov/genomes/all/GCA/001/433/935/GCA_001433935.1_IRGSP-1.0/, 10 October 2015) using STAR [95].

### 4.6. Differentially Expressed Genes (DEGs) Screening and Data Analyses

Differentially expressed genes (DEGs) from all samples were identified using DESeq2 with |log_2_fold change| ≥ 1 and a false discovery rate (FDR) < 0.05, with the FDR adjusted by the Benjamini–Hochberg method to account for multiple hypothesis testing [96]. Statistical methods of enrichment analyses generally use hypergeometric tests to identify the GO term or KEGG pathway where differential genes are significantly enriched relative to all annotated genes. The clusterProfiler software was used for DEG enrichment analyses with *p*-value < 0.05 [97].

### 4.7. RNA-Seq Data Validation by Quantitative Real-Time PCR (qRT-PCR)

Total RNA was extracted from the root tissue using the StarSpin Plant RNA Kit (GenStar, Beijing, China) according to the manufacturer’s instructions. DEPC water dissolved the extracted total RNA (1 µg) and then prepared it for cDNA synthesis using StarScript III All-in-one RT Mix with gDNA Remover (GenStar, China). The qRT-PCR was performed using 2×RealStar Fast SYBR qPCR Mix (GenStar, China) on the CFX Connect Real-Time PCR Detection System (Bio-Rad, Hercules, CA, USA). All reactions were performed in three biological replicates. The relative expression levels were calculated using the 2^−ΔΔCT^ [98]. The qRT-PCR primers are listed in Table S2.

### 4.8. Weighted Gene Co-Expression Network Analysis (WGCNA)

After filtering genes or samples with low quality (FPKM < 1), all expressed genes were prepared to construct a co-expression network using the WGCNA R package (version: 1.69) [99]. By adjusting a soft threshold (β) to 10, the transcript levels of these genes were converted into a similarity matrix and transformed into a topological overlap matrix (TOM). The dynamic tree cut method was used to identify co-expressed gene modules of the gene dendrogram, with a height cutoff of 0.25 for merging highly correlated modules. The Pearson test was employed to correlate module eigengenes and the differential phenotype for drought stress, and individual modules at *p* < 0.05 were considered to be significantly correlated with the drought-tolerant phenotype. The networks of hub genes and the top 50 genes with the highest connectivity were visualized using Cytoscape software (3.10.0).

### 4.9. Gene Cloning and Plasmid Construction

PCR amplification of candidate gene coding regions was performed using the 2×SuperNova PCR Mix (GenStar). Gene-specific primers with enzyme digestion sites and homologous arms were designed using Snapgene 6.0.2 and oligo.7 software (v7.0.6.0). The primers for gene cloning were listed in Table S2. Upon purification by gel recovery (Gel Extraction Kit, Omega), the purified PCR amplification products were then ligated into the double-enzyme (*BamH*I and *EcoR*I) digested pYES2 vector using the EZ-HiFi Seamless Cloning kit (GenStar). The recombinant vectors were transformed into *E. coli* DH5α competent cells following the manufacturer’s instructions. Positive clones were selected on LB/ampicillin plates and then verified by PCR and Sanger sequencing (Tsingke, Beijing, China). The correct positive recombinant plasmid was used for yeast transformation.

### 4.10. Yeast Transformation and Drought Sensitivity Testing

The recombinant plasmids were transformed into *S. cerevisiae* strain BY4741 using the LiAc/PEG method. Positive transformants were selected on SD-Ura medium and then cultivated in liquid SG-Ura medium. The drought tolerance of the yeast strains expressing the target genes was evaluated by spotting serial dilutions onto SG-Ura plates supplemented with 135 mM PEG3350.

### 4.11. Data Analyses

The SPSS 25.0 program (USA) was employed for variance analyses using the one-way ANOVA method. The bar diagram was prepared using Excel and GraphPad Prism (Version 8.0) software. Heatmaps were generated by using the chiplot online tool (https://www.chiplot.online/heatmap.html, 10 May 2026).

## 5. Conclusions

Compared with the drought-sensitive genotype WAB, the drought-tolerant IR65 maintained a more robust root system with enhanced ROS detoxification and osmotic regulation capabilities and showed DEGs associated with drought-tolerance pathways that responded strongly to drought stress. Combining transcriptomic data with WGCNA, significant co-expression modules were identified. Heterologous expression in yeast further validated the DEGs within these modules as potential targets for developing elite rice germplasm in water-limited fields.

## Figures and Tables

**Figure 3:**
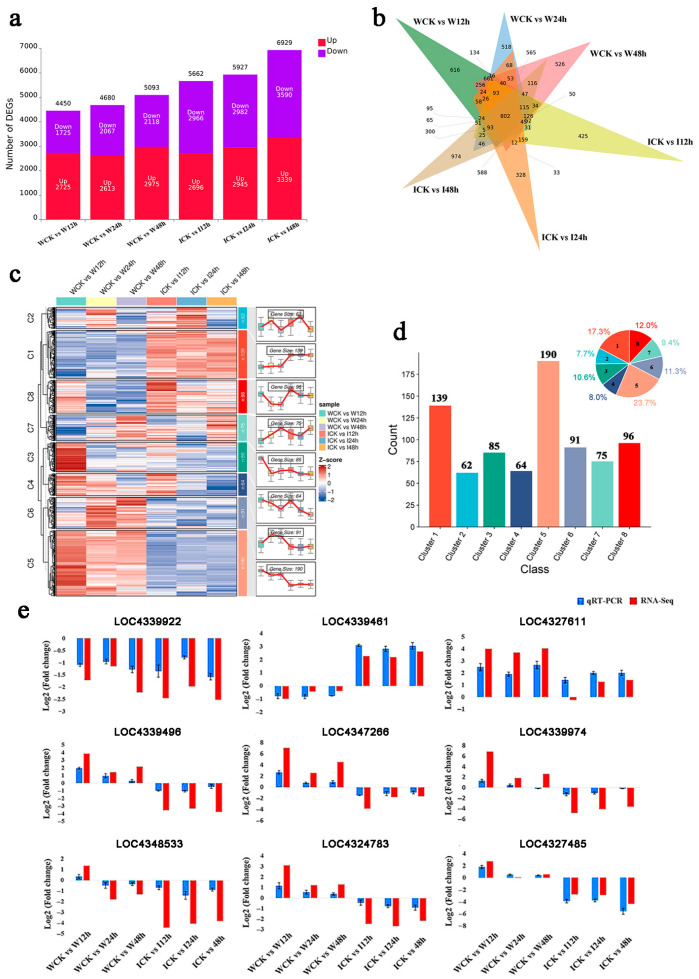
DEG variation in rice genotypes WAB and IR65 at different drought timepoints. (**a**) Up- and down-regulated DEGs in WAB and IR65; (**b**) Venn diagram of DEGs for WAB and IR65; (**c**) Clustering of DEGs; (**d**) Count and percentage of DEGs in each class; (**e**) RNA-seq validated by qRT-PCR. Transcript levels of 9 DEGs and the corresponding expression data of RNA-seq. The gene expression level of RNA-seq is presented as Log_2_(fold change), and the fold change is based on FPKM values of the drought stress group compared to the CK. The gene expression level of qRT-PCR is presented as Log_2_(fold change = 2^−^^ΔΔCT^). The bars represent SD (*n* = 3).

## Data Availability

The Illumina sequencing data have been uploaded to the National Center of Biotechnology Information (NCBI) database under accession number PRJNA1447235.

## Supplementary Materials

The following supporting information can be downloaded at https://www.mdpi.com/article/10.3390/plants15111591/s1. Table S1: Information on the summary of RNA-seq. Table S2: Information on primers for gene cloning and qRT-PCR. Figure S1: Correlation analyses of different samples based on gene expression. (a) FPKM density profile; (b) FPKM box diagram; (c) principal component analyses; (d) correlation heat maps. Figure S2: The GO enrichment analyses of WAB. Figure S3: The GO enrichment analyses of IR65. Figure S4: The KEGG analyses of WAB. Figure S5: The KEGG analyses of IR65.
