# Supplementary figures and images for: Transcriptomic Analyses and Weighted Gene Co-Expression Network Analysis (WGCNA) Identify Key Drought-Responsive Genes in Rice Roots (Oryza sativa L.) Under PEG Treatment
Source: Plants (Basel). 2026 May 22;15(11):1591. doi: 10.3390/plants15111591 (PMC13259270; doi:10.3390/plants15111591)

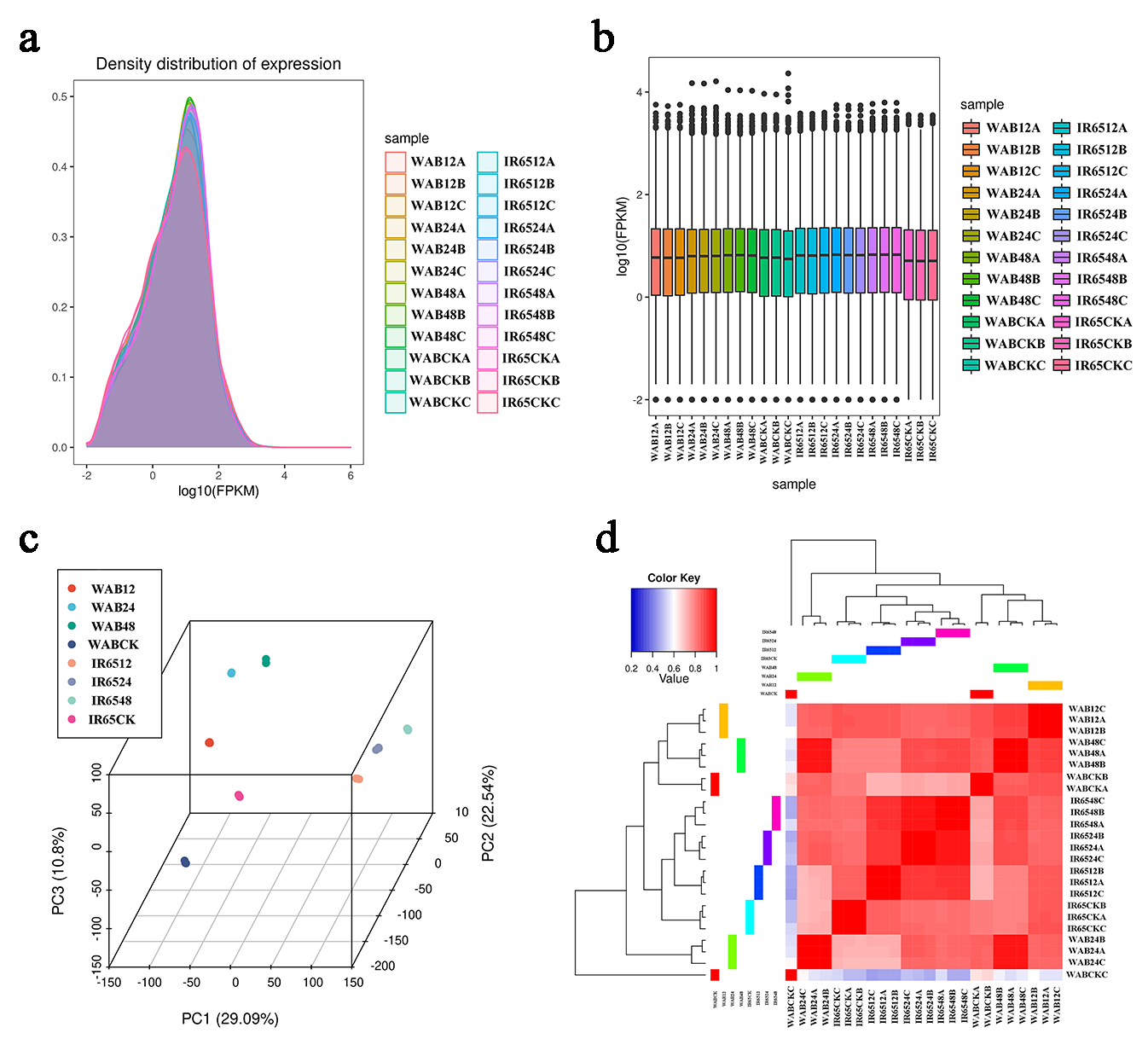

Supplement: Supplementary file 1 [file plants-15-01591-s001.zip › Figure S1.tif]

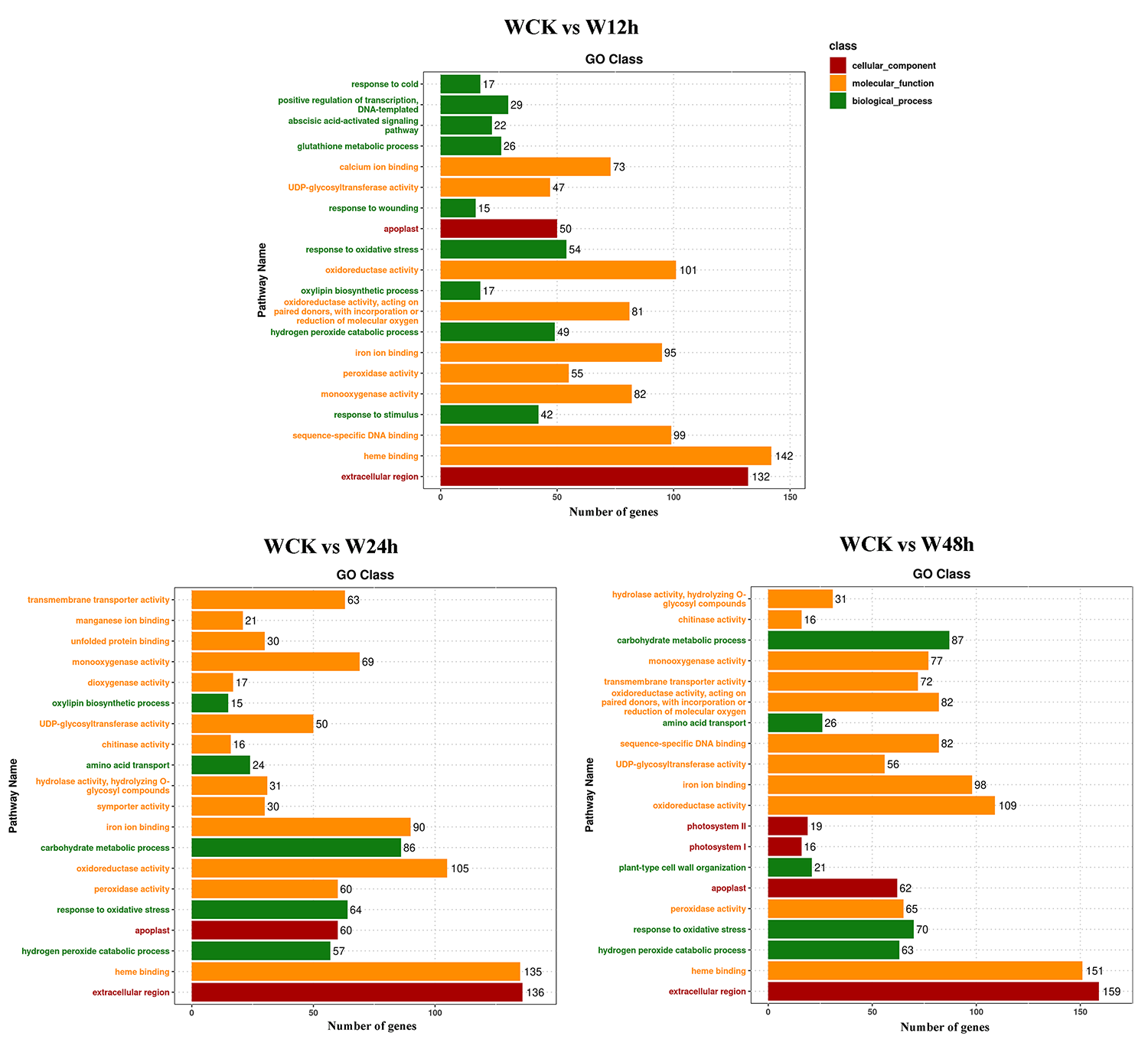

Supplement: Supplementary file 1 [file plants-15-01591-s001.zip › Figure S2.tif]

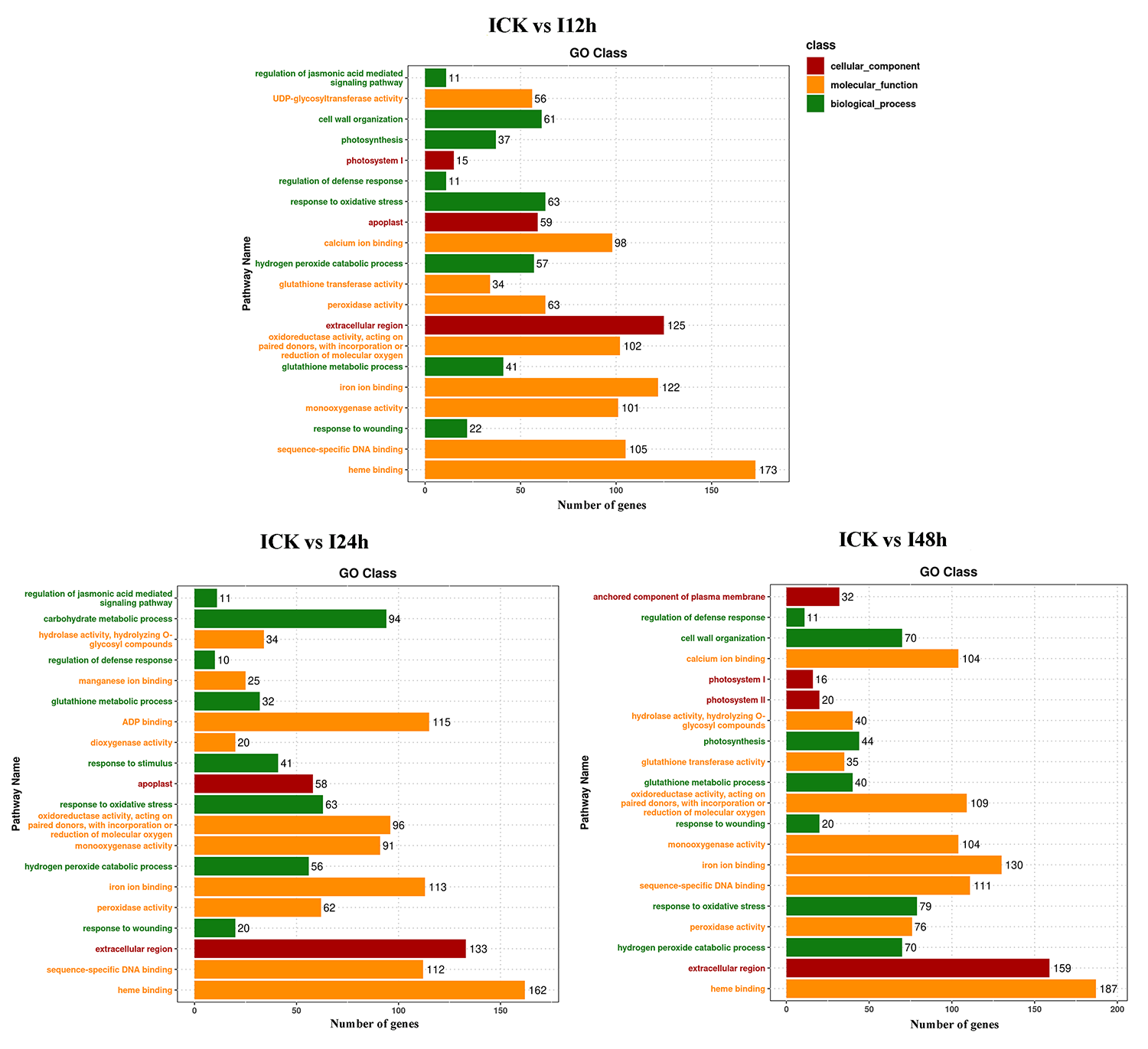

Supplement: Supplementary file 1 [file plants-15-01591-s001.zip › Figure S3.tif]

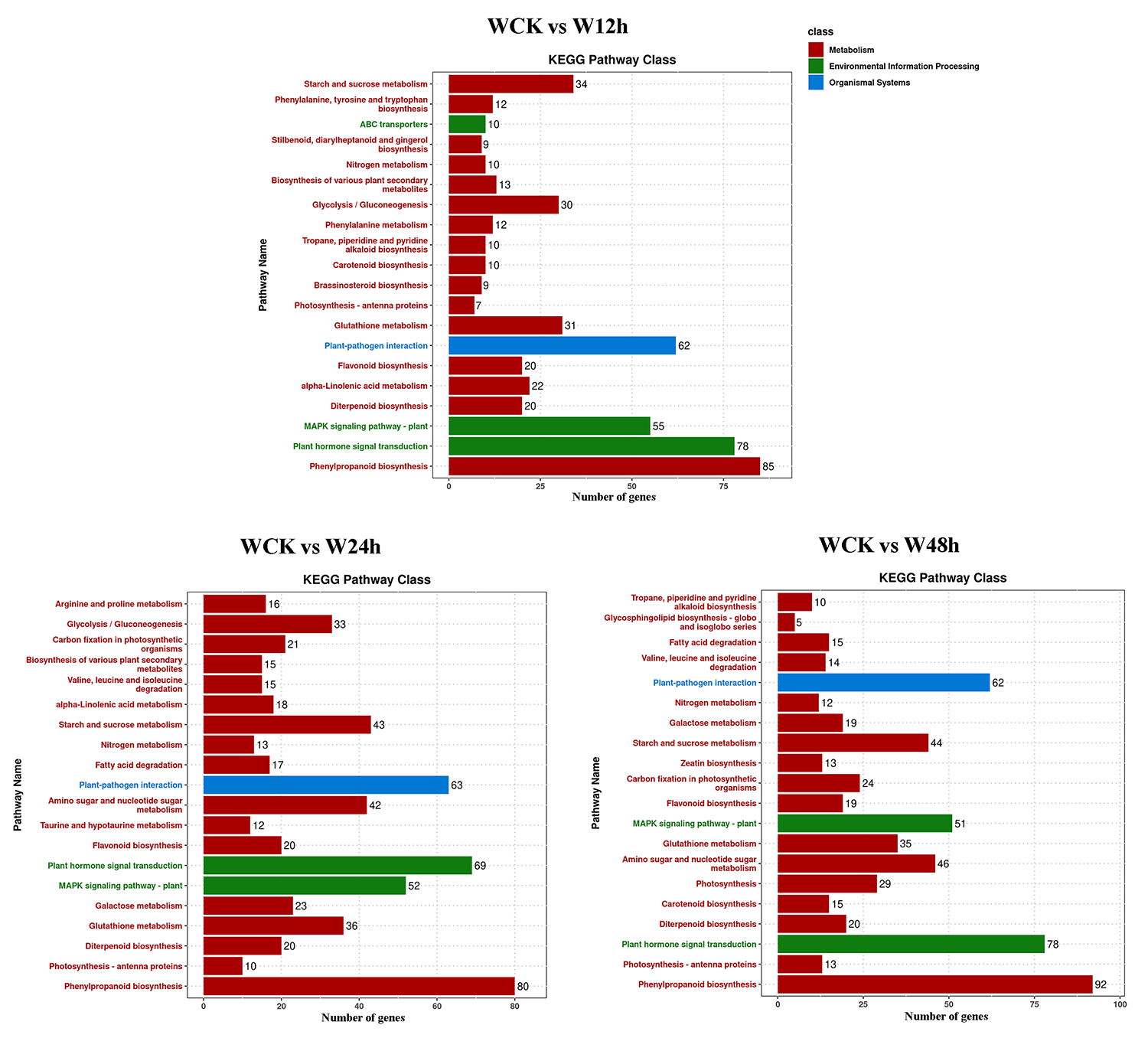

Supplement: Supplementary file 1 [file plants-15-01591-s001.zip › Figure S4.tif]

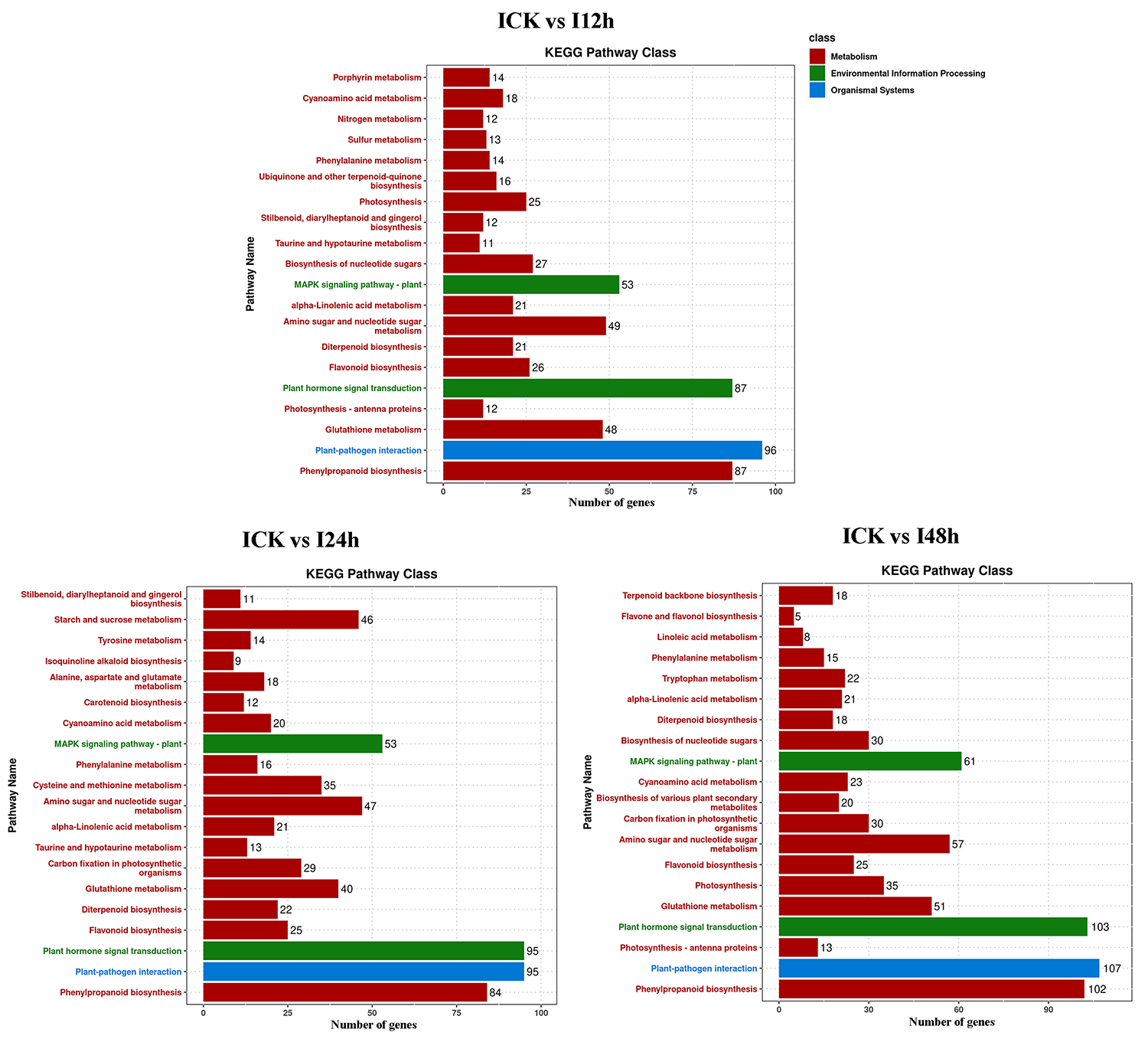

Supplement: Supplementary file 1 [file plants-15-01591-s001.zip › Figure S5.tif]
